# Omentopexy Effect on the Upper Gastrointestinal Symptoms and the Esophagogastroduodenoscopy Findings in Patients Undergoing Sleeve Gastrectomy

**DOI:** 10.1007/s11695-022-05995-0

**Published:** 2022-03-23

**Authors:** Amir K. Abosayed, Mohamed Saber Mostafa

**Affiliations:** Department of General Surgery, Kasr Alainy Hospital, Faculty of Medicine, Cairo University, ELmanial Cairo, Egypt

**Keywords:** Obesity, Laparoscopic sleeve gastrectomy, Omentopexy, Upper GIT symptoms

## Abstract

**Background:**

Laparoscopic sleeve gastrectomy (LSG) has gained acceptance worldwide. However, SG has its own complications that need a specialized management. Omentopexy is a technique in which the sleeved part of the stomach is fixed to the greater omentum.

**Aim of the Study:**

The present work aimed to investigate the potential effect of omentopexy on the upper GIT disturbances in patients with severe obesity and undergoing LSG.

**Patients and Methods:**

This study included patients who were recruited for LSG in our institution from June 2019 to October 2020. Patients having no upper GIT symptoms, no esophagogastroduodenoscopy (EGD) GERD signs, and no hiatus hernia were eligible for the study. Patients were randomly enrolled into the omentopexy group (underwent LSG with omentopexy) and the non-omentopexy group (underwent LSG only). Patients were followed up 1 month, 3 months, and 1 year after the operation. EGD was performed at the 1-year follow-up.

**Results:**

Forty-five patients constituted the omentopexy group and forty-six constituted the non-omentopexy group. Omentopexy was associated with significant reduction in the early post LSG upper GIT symptoms, and less EGD evident reflux esophagitis at the 1-year follow-up (statistically non-significant).

**Conclusion:**

The current work adds a new evidence of the omentopexy benefits in patients undergoing sleeve gastrostomy, with an overall better outcome in regard to the upper GIT upset and GERD compared to LSG alone.

**Graphical abstract:**

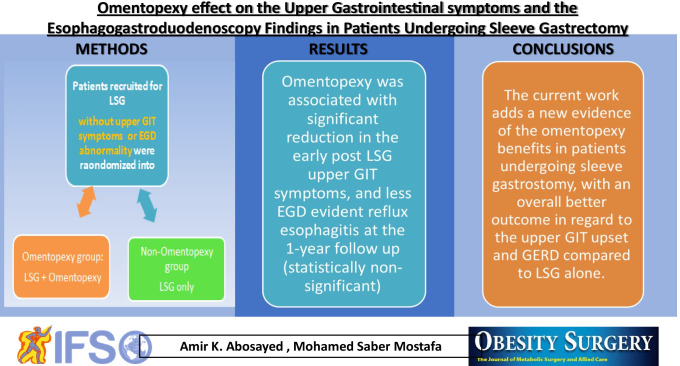

## Introduction

Obesity has been widely prevalent and even has recently considered a pandemic, with several impacts on the human life [1]. Therefore, bariatric surgery procedures have acquired an outstanding importance as the most effective treatment in patients with severe obesity, among whom the lifestyle modification and the medical treatments do not give satisfactory outcome [2]. Bariatric surgery has shown excellent results, not only in reducing weight, but also in amelioration of the obesity-associated comorbidities [3]. Of the bariatric surgery procedures, laparoscopic sleeve gastrectomy has gained acceptance worldwide. It is remarked by the technical simplicity, the preservation to the gastrointestinal (GIT) anatomy, the absence of an anastomosis step, and the excellent outcome as evolved from several research studies [4]. Nevertheless, laparoscopic sleeve gastrectomy (LSG) has been reported to be associated with upper GIT disorders, including gastro-esophageal reflux disease (GERD), with annoying symptoms such as nausea, vomiting, and fluid intolerance [5]. Moreover, staple line bleeding and gastric leakage are of the most common serious complications encountered after LSG [6]. Thus, several modification techniques have been adopted to reduce the LSG-associated complications and to get the surgery benefits as much as possible. Omentopexy is one of these modifications. It is a technique in which the sleeved part of the stomach is fixed to greater omentum [7]. It is presumed that the stomach fixation through the omentopexy amends the gastric twist, and hence precludes the functional stenosis [8]. Omentopexy is thought to be alleviating the LSG-related GERD, food intolerance, and leak [9]. However, data about the efficacy of this technique is still scarce. The present work aimed to investigate the potential effect of omentopexy on the upper GIT disturbances in patients with severe obesity and undergoing LSG.

## Patients and Methods

This is a prospective randomized controlled study that was conducted at Kasr Al-Ainy Hospital during the period from June 2019 to October 2020. The study included patients recruited for bariatric surgery at our institution. Patients’ eligibility criteria for bariatric surgery were being adult, having a BMI higher than 40 kg/m^2^ or 35 kg/m^2^ with comorbidities, and being fit for surgery under general anesthesia. The patients selected for LSG, as recommended by the department standards, were included in the study. Institutional research ethics committee approval was obtained before starting the study. The study was performed in accordance with Helsinki declaration, and an informed written consent was obtained from each patient.

All patients underwent complete history taking, medical examination and routine laboratory, and radiological investigations. Preoperative esophagogastroduodenoscopy (EGD) examination was performed for all patients. Patients with history suggestive of GERD and or abnormal EGD examination (such as esophagitis or hiatus hernia) were excluded from the study. Upper GIT symptom evaluation was performed based on Rome III criteria [10].

### Randomization of the Patients

The included patients were randomized into two groups at a ratio of 1:1. Randomization was performed blindly by a physician who did not participate in the study. Opaque sealed envelopes containing sequential numbers were given to the study patients, according to which each patient was enrolled to one of the two groups.

### Surgical Technique

Patients of both groups underwent the preoperative preparation and had the surgery performed under general anesthesia. The surgery was performed as standardized via 5-port technique after induction of pneumoperitoneum. The greater curvature vascularity division was carried out using an advanced bipolar sealing device (*LigaSure*), beginning at about 6 cm from the pylorus and proceeding till the angle of His. A 36-Fr calibrated bougie tube was inserted trans-orally and positioned strictly against the lesser curve, and then the sleeve line was stapled using a linear stapler. Methylene blue test was performed, and the excised stomach was removed.

**Fig. 1:**
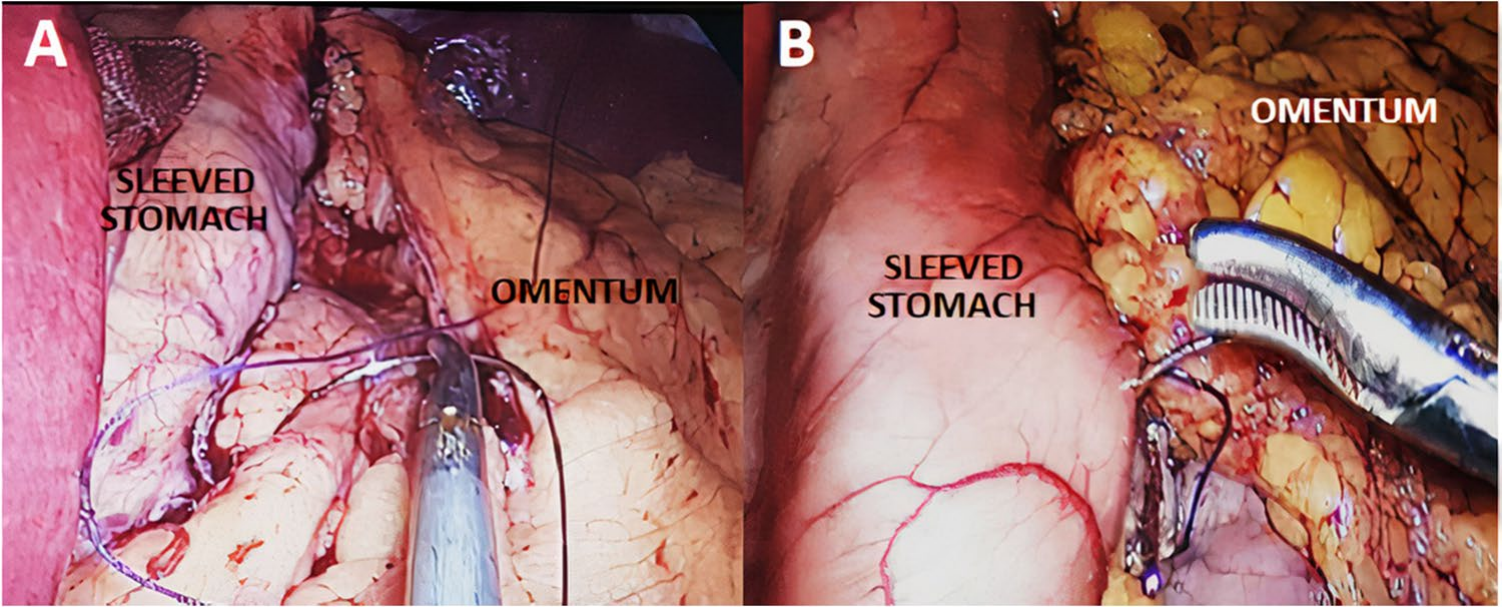
Omentopexy sutures: **A** beginning suturing the greater omentum to the staple line and **B** near the end of the procedure

For patients in the omentopexy group, the sleeved stomach was fixed to the free edge of the greater omentum using vicryle 2–0 sutures, beginning at 1 cm from the gastroesophageal junction and proceeding along the entire staple line, with the sutures 1-cm apart (Fig. 1).

Full thickness suturing of the omentum was performed in a continuous manner close to the site where it was separated from the stomach to avoid injuring the major omental vessels. In most of the sleeved stomach, suturing was seromuscular with full invagination of the staple line within the omentum, to restore the preoperative normal configuration as far as possible. Below the level of the incisura angularis, where the tissue became thicker, full thickness suturing was done.

Patients found having hiatus hernia during the operation had the hernia repaired and were excluded from the study.

### Postoperative Management

All patients were encouraged for early movement, and allowed for fluids 2 h after the operation, which was gradually transformed into solid diet over a period of 2–3 weeks. Postoperative prophylactic anticoagulants and proton pump inhibitors were prescribed for 2 weeks and 3 months, respectively. The operative events were recorded, and before discharge, the patients were informed to seek medical advice at the outpatient or the department whenever they encounter any adverse event.

Patients had follow-up examinations 1 month, 3 months, and 1 year after the surgery, during which they underwent complete history taking and clinical examination. After 3 months of surgery, the patients who were still having upper GIT disturbance symptoms were continued on proton pump inhibitors till 1 year postoperatively.

At 1 year postoperatively, the study patients underwent another EGD examination.

### The Study Outcomes

The primary outcome of this study was the differences between both groups in the postoperative upper GIT clinical and EGD findings, and the secondary outcome was the difference in the operative events and other complications.

### Statistical Methods

Data were analyzed using the statistical software SPSS (IBM Corp., Armonk, NY, USA) version 22. After testing data normality, Mann–Whitney test and Student *t*-test were used to compare numerical data accordingly. Chi-square and Z score for proportion tests were used to compare categorical data as appropriate. Differences were considered statistically significant when *p* values were less than 0.05.

## Results

The preliminary number of patients enrolled to each group was 50. After exclusion of cases who were found intraoperatively having hiatus hernia and the cases that dropped out after 1 year of the operation, forty-five patients constituted the omentopexy group, and forty-six patients constituted the non-omentopexy group.

The study patients’ age ranged from 20 to 60 with a mean of 36 ± 10.5 years. They were predominantly females (82.4%), and had a mean weight of 123.7 ± 17.6 kg and a mean BMI of 46.7 ± 4.9 kg/m^2^. The associated comorbidities were hyperlipidemia, obstructive sleep apnea, diabetes mellitus, and hypertension, with prevalence of 100%, 38.5%, 37.4%, and 48.4%, respectively. In the omentopexy group, the mean age was 34.5 ± 10.7, the females’ percentage was 88.9%, the mean weight was 123.4 ± 18.3, and the mean BMI was 46.9 ± 5.8 kg/m^2^. The comorbidities rates were 100%, 44.4%, 40%, and 46.6% for hyperlipidemia, sleep apnea, diabetes mellitus, and hypertension, respectively. In the non-omentopexy group, the mean age was 37.4 ± 10.2, the females’ percentage was 76.1%, the mean weight was 123.7 ± 17.1 kg, and the mean BMI was 46.5 ± 3.8 kg/m^2^. The comorbidity rates were 100%, 32.6%, 34.8%, and 50% for hyperlipidemia, sleep apnea, diabetes mellitus, and hypertension, respectively. No significant differences were found between the omentopexy and the non-omentopexy groups in the age, the female percentage, the weight, the BMI, or the comorbidities prevalence (Table 1).

**Table 1:** Baseline data of the study patients

		Omentopexy group (*n* = 45)	Non-omentopexy group (*n* = 46)	p
		Mean ± SD	Mean ± SD	
		Median (min–max)	Median (min–max)	
**Age** (years)		34.5 ± 10.7 33 (20–60)	37.4 ± 10.2 36 (22–55)	0.1^U^
**Weight** (kg)		123.4 ± 18.3 125 (100–170)	123.7 ± 17.1 122 (90–155)	0.72^U^
**BMI** (kg/m^2^)		46.9 ± 5.8 46 (40–60)	46.5 ± 3.8 45.5 (40–55)	0.51^U^
		**n** (%)	**n** (%)	
**Gender**	Female	40 (88.9)	35 (76.1)	0.12^X^
	Male	5 (11.1)	11 (23.9)	
**Comorbidities**	Hyperlipidemia	45 (100)	46 (100)	–
	OSA	20 (44.4)	15 (32.6)	0.25^Z^
	Diabetes mellitus	18 (40)	16 (34.8)	0.61^Z^
	Hypertension	21 (46.6)	23 (50)	0.75^Z^

^U^Mann-Whitney test

^X^Chi-square test

^Z^Z score for proportion

The mean operative time was 64.1 ± 13 min. The hospital stay ranged from 1 to 4 days, with a mean of 1.08 ± 0.4 days. Statistically significant higher operative time was needed in the omentopexy group (the omentopexy group had a median time of 70 min compared to a median of 55 min in the non-omentopexy group), while no statically significant difference was noted in the hospital stay length (the median times in omentopexy and non-omentopexy groups were 1 and 1.2 days, respectively) (Table 2).

**Table 2:** Operative data of the study patients

	Omentopexy group (*n* = 45)	Non-omentopexy group (*n* = 46)	p
	Mean ± SD	Mean ± SD	
	Median (min–max)	Median (min–max)	
**Operative time** (minutes)	58.4 ± 10.6	56.4 ± 10.2	< 0.001^U*^
	70 (55–100)	55 (40–80)	
**Hospital stay length** (days)	1.02 ± 0.15	1.13 ± 0.5	0.18^U^
	1 (1–2)	1.22 (1–4)	
	***n*** (%)	***n*** (%)	
**Leakage**	1 (2.2)	2 (4.3)	0.57^Z^
**Bleeding**	0 (0)	2 (4.3)	0.16^Z^

^*^Statistically significant

^U^Mann-Whitney test

^Z^Z score for proportion

Postoperative bleeding was encountered in 2 (2.2%) patients in the non-omentopexy group, where conservative treatment, with resuscitation and blood transfusion, was sufficient to control the condition. Postoperative leakage was suspected clinically in 3 cases, and then confirmed with computed tomography (CT) examination. Two cases responded to the conservative medical treatment, as demonstrated in the follow-up CT, while one case required management with gastric stent insertion, and this was in the non-omentopexy group (Table 2).

One-month postoperatively, 16.5% of the patients gave a history of experiencing one or more of the following symptoms: nausea, vomiting, fluid intolerance, heart burn, dyspepsia, dysphagia, regurgitation, and chest pain, at least once per week. The most common of which was the nausea symptom (9.9%). It was found that 8.8% of the patients experienced three or more of these symptoms. Statistically significant difference was observed between both groups in the incidence of the early postoperative upper GIT symptoms, where an incidence of 6.6% was found in the omentopexy group compared to 26.1% in the non-omentopexy group. Analysis of each symptom frequency revealed significantly lower incidence of fluid intolerance and chest pain (*p* = 0.023 and 0.043, respectively). The incidences of nausea, heart burn, and regurgitation were also lower in the omentopexy group (4.4% vs. 15.2%, 2.2% vs. 13%, and 2.2% vs. 13%, respectively). However, the differences were a bit from statistical significance (*p* = 0.085, 0.053, and 0.053, respectively) (Table 3).

**Table 3:** Comparison between the study groups in the upper GIT symptoms

		**Omentopexy group** (*n* = 45)	**Non-omentopexy group** (*n* = 46)	**p**
		***n*** (%)	***n*** (%)	
**1-month symptoms**	No symptoms	42(93.4)	34 (73.9)	0.037^X^*
	Less than 3	2 (4.4)	5 (10.9)	
	3 or more	1(2.2)	7 (15.2)	
	Nausea	2 (4.4)	7 (15.2)	0.085^Z^
	Vomiting	1 (2.2)	2 (4.3)	1 (2.2)
	Fluid intolerance	0 (0)	5 (10.9)	0.023^Z^*
	Heart burn	1 (2.2)	6 (13)	0.053^Z^
	Dyspepsia	1 (2.2)	2 (4.3)	0.57^Z^
	Dysphagia	1 (2.2)	1 (2.2)	0.99^Z^
	Regurgitation	1 (2.2)	6 (13)	0.053^Z^
	Chest pain	0 (0)	4 (8.7)	0.043^Z^*
**3-month symptoms**	No symptoms	42 (93.3)	41 (89.1)	0.34^X^
	Less than 3	2 (4.4)	1 (2.2)	
	3 or more	1 (2.2)	4 (8.7)	
	Nausea	1 (2.2)	2 (4.3)	0.57^Z^
	Vomiting	0 (0)	0 (0)	–
	Fluid intolerance	0 (0)	0 (0)	–
	Heart burn	2 (4.4)	4 (8.7)	0.41^Z^
	Dyspepsia	1 (2.2)	1 (2.2)	0.99^Z^
	Dysphagia	1 (2.2)	1 (2.2)	0.99^Z^
	Regurgitation	1 (2.4)	4 (8.7)	0.18^Z^
	Chest pain	0 (0)	2 (4.3)	0.16^Z^

^*^Statistically significant

^U^Mann-Whitney test

^X^Chi-square test

^Z^Z score for proportion

Three months postoperatively, only 8.8% of patients remained having upper GIT disturbance symptoms. Of which, 5.5% were having 3 symptoms or more. Despite higher percentage of symptomatic patients in the non-omentopexy group (10.9% compared to 6.7% in the omentopexy group), the difference between both groups was statistically non-significant. No cases of vomiting or fluid intolerance were still encountered. Higher incidences of nausea, heart burn, regurgitation, and chest pain were encountered in the non-omentopexy group (4.3% vs. 2.2%, 8.7% vs.4.4%, 8.7% vs. 2.4%, and 4.3% vs. 0%, respectively), yet with no statistical significance (Table 3).

At the 1-year postoperative follow-up, the patients had a mean weight of 77.7 ± 10.2 kg, a mean BMI of 28.9 ± 2.8 kg/m^2^, and a mean percentage of total weight loss (%TWL) of 36.9 ± 3.6%. Both groups were comparable in the postoperative weight measures (Table 4).

**Table 4:** One-year postoperative data of the study patients

		**Omentopexy group** (*n* = 45)	**Non-omentopexy group** (*n* = 46)	**p**
		Mean ± SD	Mean ± SD	
		Median (min–max)	Median (min–max)	
**Weight** (kg)		77.9 ± 9.6	77.5 ± 10.8	0.87^ T^
		75 (65–103)	78 (60–102)	
**BMI** (kg/m^2^)		29.1 ± 2.9	28.6 ± 2.6	0.47^U^
		29 (25–36)	28 (25–33)	
**%TWL**		36.6 ± 3.8	37.2 ± 3.4	0.38^ T^
		36.9 (28–43)	37.1 (30–43)	
		***n*** (%)	***n*** (%)	
**Comorbidities**	Hyperlipidemia	4 (8.8)	3 (6.5)	0.42^Z^
	OSA	9 (20)	13 (28.3)	0.36^Z^
	Diabetes mellitus	2 (4.4)	3 (6.5)	0.66^Z^
	Hypertension	5 (11.1)	6 (13)	0.78^Z^

^U^Mann-Whitney test

^T^Student *t-*test

^Z^Z score for proportion

There was a remission of the patients’ comorbidities at variable rates. Hyperlipidemia was found in 7 (7.7%) cases only, with a remission rate of 92.3%. The remission rates of OSA, diabetes mellitus, and hypertension were 74.3, 85.3, and 75%, respectively (Table 4).

The EGD examination at the 1-year postoperative follow-up revealed that 9.9% of patients had reflux esophagitis, of which 7.7% were of grade A and 2.2% were of grade B. Higher percentage of reflux esophagitis was observed in the non-omentopexy group (15.2% compared to 4.4% in the omentopexy group). Moreover, no cases of grade B esophagitis were found in the omentopexy group, compared to 4.4% incidence in the non-omentopexy group. However, the difference did not reach the significance level (Table 5).

**Table 5:** Comparison between groups in the postoperative EGD examination

	**Omentopexy group** (*n* = 45)	**Non-omentopexy group** (*n* = 46)	**p**
	***n*** (%)	***n*** (%)	
**No esophagitis**	43 (95.6)	39 (84.8)	0.18^X^
**Grade A esophagitis**	2 (4.4)	5 (10.9)	
**Grade B esophagitis**	0 (0)	2 (4.3)	

^X^Chi-square test

## Discussion

The effectiveness and safety of LSG were described by several authors. However, complications specific to LSG were also described [6]. The most serious of which are the bleeding and leakage from the staple line, in addition to the gastric volvulus [8]. Despite being not as serious as leakage and bleeding, the post LSG upper GIT disturbances are annoying the patients, having negative impacts on their quality of life, and may even be associated with more risky conditions [11].

The GERD complications associated with sleeve gastrectomy have been controversial. Theoretically, the reduced gastric mass should minimize the production of gastric acid; however, it has been reported by several authors that LSG is related to GERD aggravation. This was attributed to the disruption of the His angle, the hypotonic lower esophageal sphincter, the conversion of a large compliant stomach to a narrow high pressure tube, or the decreased gastric emptying [12, 13].

Omentopexy implies fixating the greater omentum to the sleeved stomach staple line [7]. The benefits of adding the omentopexy step to the LSG procedure is still in debate.

Omentopexy has not been a standardized procedure yet. Its technique varies among different studies. In the study of Sharma et al. [14], the authors performed 2–4 sutures proximal to the incisura and 1 suture distally at the end of the staple line [15]. Pilone et al. [15] placed a synthetic sealant layer on the sutures and covered it by an omentum flap. Abou-Ashour et al. [16] placed 4–6 sutures fixing the greater curvature axis. Batman et al. performed omentopexy by suturing the omentum to the greater curvature along the staple line with V-Loc sutures [17]. In the current study, we applied continuous suturing of the greater omentum to the greater curvature alongside the entire staple line, in an attempt to restore the preoperative anatomical assembly as far as possible, to get the maximum stabilization of the posterior wall of the stomach.

The present study showed that a statistically higher operative time was needed in the omentopexy group, this is simply explained by the added omentopexy step. However, we think that this difference will gradually diminish as soon as the procedure becomes familiar to the performing surgeons. In harmony with our study, omentopexy was reported to be associated with minimal operative time prolongation [18]. In Labib study [19], the difference was about 16 min, while in Abou-Ashour study, the difference was only 5 min [16]. Higher differences were described by Nosrati et al. also (20 min) [20] and Sabry and Qassem (30 min) [21].

The present study is in congruence with most of the related literature that describes statistically non-significant difference in the hospital stay length. The most recent meta-analysis of Zarzycki et al. [9] reported no significant difference between both groups in the hospital stay duration. Labib [19] and Afaneh et al. [22] reported the same findings. In variance with our findings, Pilone et al. [15] and Sabry and Qassem [21] found a significant longer hospital stay in the non-omentopexy group. Nevertheless, the difference was clinically negligible in the later study (6 h).

The present study showed non-significant higher incidence of postoperative bleeding in the non-omentopexy group. In agreement with our study, several studies reported that omentopexy was associated with reduced bleeding incidence, either significantly [21] or non-significantly [15, 19].

Likewise, non-significant higher incidence of leakage was found in the non-omentopexy group. This is similar to Pilone et al. findings, who found fewer cases of leakage in the omentopexy group, yet, with no statistical significance [15].

Reduced bleeding and leakage incidence in the omentopexy group is likely explained by the omentum characteristics that help to seal the oozing surfaces. It was also assumed that the lessened twisting and kinking associated with omentopexy reduce the incidence of proximal leak.

At the 1-year postoperative follow-up, there was an excellent outcome regarding weight loss and comorbidity remission, with comparable outcome in the two groups. The present work confirmed the previously described outcome of LSG [23].

Regarding the primary outcome of this study, at 1-month postoperatively, significantly lower frequency of the upper GIT upset symptoms was observed in the omentopexy group (6.7% vs. 19.6%).

In this study, the upper GIT symptoms lessened after 3 months. This may be prompted in part by the better gastric compliance and the effect of proton pump inhibitors. Although the symptoms were still lower in the omentopexy group, the statistical significance faded away.

It was suggested by Arslan et al. [8] that adding omentopexy to sleeve gastrectomy stabilizes the posterior gastric wall. Hence, it can impede the twisting of the stomach, which is implicated to be the functional cause of gastric stenosis.

In consistency with the current study, Filho et al. [24] and Abou-Ashour [16] declared that LSG with omentopexy was associated with GERD clinical improvement. However, other researchers found that omentopexy had no effect on the GERD condition [11, 20]. This discrepancy in findings may be explained by variances in the investigated cohort, the study design, or the technique of LSG and omentopexy.

This is the first study, to the best of our knowledge, assessing the difference between omentopexy and non-omentopexy groups in the EGD findings 1 year after the surgery. Nine cases (9.9%) were diagnosed with esophagitis after 1 year in this study. In the same context, Braghetto et al. [12] reported an esophagitis incidence after LSG of 15.5%. Similarly, Tai et al. [25] and Viscido et al. [5] found an increase in cases of esophagitis after the operation.

In the present study, the non-omentopexy group showed higher number and higher grade of esophagitis. Despite being statistically non-significant, this difference could be clinically significant, and the statistical significance could occur with a larger sample study.

In consistency with our findings, it was proposed that the loss of gastric fixation could yield inappropriate positioning of the sleeved stomach, with subsequent permanent GERD [26].

The present study is supporting the positive impact of omentopexy on the post LSG upper GIT disorders, shortly after the operation (as manifested in less nausea, vomiting, fluid intolerance, dyspepsia, and GERD symptoms), and after 1 year (in the form of less evident reflux esophagitis). However, larger long-term studies are still needed to obtain more consolidated conclusion.

## Strength and Limitations

The present study is strengthened by its prospective randomized controlled design, by selecting a cohort that was originally free of GERD symptoms and esophageal pathologic changes, and by adding the EGD to the patients’ evaluation. However, the study is limited by the relatively small sample size, and non-using of the esophageal function tests to assess the study patients.

## Conclusion

The current work adds a new evidence of the omentopexy benefits in patients undergoing sleeve gastrostomy, with an overall better outcome in regard to the upper GIT upset and GERD compared to LSG alone.
